# Unexpected Diagnosis in a Cutaneous Tumoral Lesion: Primary Cutaneous Leg-Type B-Cell Lymphoma

**DOI:** 10.7759/cureus.89416

**Published:** 2025-08-05

**Authors:** Ruth P Sotelo Loeza, Axel R Marquez-Nuñez, Rosalinda Peñaloza Ramirez, Cinthia F Castro Carcaño, Juan E Abad Olmedo, Elizabeth Ramos Lopez, Eleazar Hernandez Ruiz, Regina Hernandez Hernandez Canade, Lucia Achell Nava

**Affiliations:** 1 Dermatology, Centro Medico Nacional 20 de Noviembre, Instituto de Seguridad y Servicios Sociales de los Trabajadores del Estado, Mexico City, MEX; 2 Pathology, Centro Medico Nacional 20 de Noviembre, Instituto de Seguridad y Servicios Sociales de los Trabajadores del Estado, Mexico City, MEX; 3 Pathology, Centro Medico Nacional 20 de Noviembre, Instituto de Seguridad y Servicios Sociales de los Trabajadores del Estado, Mexico City, MEX

**Keywords:** aggressive cd20-negative lymphoma, immunohistochemistry staining, mycosis fungoides (mf), primary cutaneous diffuse large b-cell lymphoma - leg type, primary cutaneous lymphomas

## Abstract

Primary cutaneous diffuse large B-cell lymphoma, leg type (PCDLBCL-LT), is an uncommon and aggressive subtype of cutaneous B-cell lymphoma, typically affecting elderly women and predominantly involving the lower extremities. Its diagnosis relies on immunohistochemical profiling and clinical presentation. We report a rare case of a 45-year-old male presenting initially with scalp and supraciliary plaques. The early diagnosis suggested tumor-stage mycosis fungoides, based on histopathology and immunophenotype (CD4+, CD20-). However, over time, the patient developed disseminated nodular lesions, with new biopsies revealing CD20+, MUM1+, BCL2+, Ki-67 70%, and MYC expression, consistent with PCDLBCL-LT. Despite multiple lines of systemic therapy, including R-CHOP (cyclophosphamide, doxorubicin, vincristine, and prednisone combined with rituximab), methotrexate, gemcitabine, platinum-based chemotherapy, and autologous stem cell transplant, the disease progressed, ultimately leading to the patient’s death. This case highlights an atypical clinical presentation of PCDLBCL-LT in a young male, initially mimicking T-cell lymphoma. The diagnostic evolution underscores the importance of repeated biopsies and immunohistochemical reevaluation in persistent or atypical cutaneous lymphoproliferative disorders. It also raises awareness of CD20-negative variants and potential coexistence with T-cell lymphomas such as mycosis fungoides.

## Introduction

Primary cutaneous diffuse large B-cell lymphoma, leg type (PCDLBCL-LT), accounts for approximately 20% of all primary cutaneous B-cell lymphomas [1]. It characteristically presents on the lower extremities of elderly female patients, with a median age of 76 years, a five-year overall survival rate of 55%, and relapse rates of up to 18% following treatment [2].

Clinically, it typically manifests as nodules or plaques on the lower limbs. Its histopathologic diagnosis and differentiation are supported by a classical immunophenotype: CD20+, BCL2+, MUM1+, variably BCL6+, CD10-, and a high Ki-67 proliferation index [3]. CD30 expression has been reported in up to 30% of T-cell lymphomas and in centroblasts and immunoblasts of 15-20% of B-cell lymphomas [4]. Additionally, there are reports of CD20-negative primary diffuse large B-cell lymphomas in 1-3% of cases [5].

The distinction between mycosis fungoides and primary cutaneous diffuse large B-cell lymphoma is essential not only for determining the appropriate treatment but also due to prognostic implications, as mycosis fungoides generally follows a more indolent clinical course and has a more favorable outcome, particularly in early stages, compared to the typically more aggressive behavior of PCDLBCL-LT.

## Case presentation

We present the case of a 45-year-old male with no significant past medical history, originally from and residing in Mexico City, Mexico. The disease began in January 2018 with the appearance of two plaques on the scalp and supraciliary region. The patient presented to the hospital a year afterward. In January 2019, an inguinal lesion biopsy was performed to determine B-cell vs. T-cell lineage, yielding a diagnosis of tumor-stage mycosis fungoides with atypical monoclonal plasmacytic proliferation. In February 2019, the patient underwent six cycles of R-CHOP (cyclophosphamide, doxorubicin, vincristine, and prednisone combined with rituximab) chemotherapy, which was completed in July 2019, resulting in a good lymph node response.

In September 2019, a biopsy of the retroauricular lesion revealed lymphoplasmacytic proliferation. Immunohistochemistry revealed CD4+, CD7-, and CD20 cells, consistent with mycosis fungoides, leading to the initiation of oral methotrexate, but without clinical improvement. In 2020, the patient received gemcitabine-based therapy, also without response, and was referred to hematology. He subsequently underwent treatment with carboplatin, etoposide, ifosfamide, and mercaptoethanol sulfonate sodium (MESNA), again with no significant response. In April 2021, a new biopsy of a lesion on the upper extremity revealed large-cell transformation with 95% CD30+ expression.

**Figure 1:**
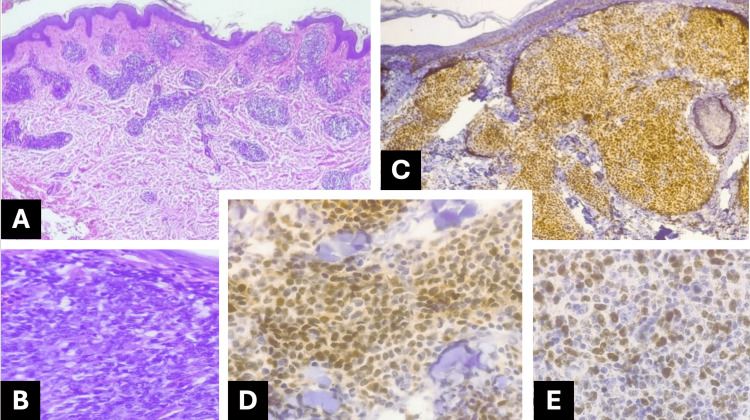
Skin biopsy from an unspecified site. The biopsy shows a dermal neoplastic infiltrate characterized by intermediate- to large-sized, monotonous, and diffuse sheets of centroblasts and immunoblasts (A). At higher magnification, the neoplastic cells include centroblasts (round, non-cleaved nuclei with vesicular chromatin and one to multiple small nucleoli) and immunoblasts (large oval nuclei with vesicular chromatin and a prominent nucleolus) (B). (Hematoxylin–eosin stain, original magnifications ×10 (A), ×40 (B)). Immunohistochemistry demonstrates strong nuclear positivity for PAX5 in neoplastic cells (C), BCL6 positivity in neoplastic cells (D), and a high proliferative index with Ki-67 expression in approximately 70% of PAX5-positive neoplastic cells (E). (Immunohistochemical stain, original magnification ×40 (C–E)).

By June 2021, the patient developed hyperpigmented plaques and masses up to 6 cm in the neck, genitalia, gluteal region, and hands, some of which were ulcerated. Inguinal lymphadenopathy was noted (up to 1.5 cm, mobile, non-tender). A computed tomography (CT) scan revealed persistent iliac and inguinal adenopathies. Radiotherapy was proposed but postponed due to nodal disease activity. In March 2023, a subsequent biopsy showed immunohistochemistry markers: CD20+++, CD5++, PAX5++, BCL2++, CD10-, MUM1+++, Ki-67: 70%, MYC+++ (Figure 1).

**Figure 2:**
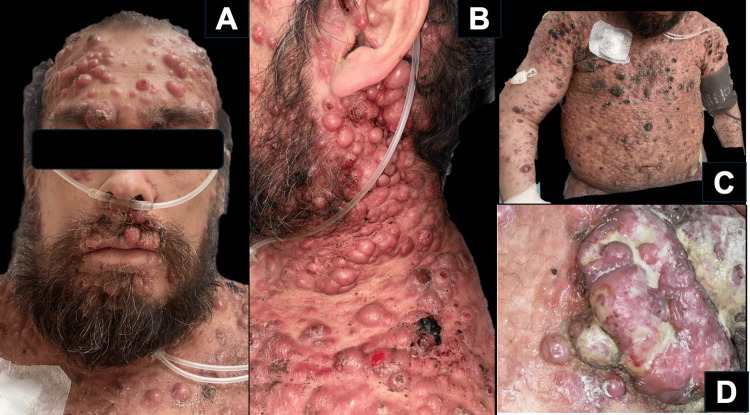
Disseminated plaques and nodules involving the head, trunk, and extremities. (A) Multiple erythematous nodules affecting the forehead, cheeks, lips, and chin. (B) Erythematous nodules involving the ear, neck, and shoulder, some appearing confluent. (C) Reddish-to-brown plaques and nodules affecting the chest, upper extremities, and abdomen. (D) Close-up of a nodules with fibrin deposition on the surface.

The final diagnosis is PCDLBCL-LT, post-germinal center phenotype. The patient was lost to follow-up during 2021-2022 due to the COVID-19 pandemic. Upon return in 2023, he presented with nodular lesions on the back and left auricle, widespread tumors, pruritus, and pain (Figure 2).

In September 2023, he underwent autologous stem cell transplantation, with an incomplete response and progressive generalized tumor growth. Disease relapse occurred in January 2024, and treatment with ibrutinib and electron beam radiotherapy was initiated, but disease progression continued, and the patient subsequently passed away.

## Discussion

DLBCL is the most prevalent type of lymphoma, representing approximately one-third of global lymphoma cases. According to the World Health Organization (WHO), a large proportion of these lymphomas are classified as not otherwise specified (NOS); however, up to 20% of cases correspond to one of the 13 recognized DLBCL variants [6]. PCLBCL-LT is composed exclusively of centroblasts and immunoblasts and typically arises on the lower extremities [7]. Clinically, it presents as erythematous to violaceous nodules measuring 2-5 cm in diameter, with or without ulceration, typically affecting the legs, and is more frequent in females aged 70-80 years [8]. Histologically, it demonstrates superficial and deep dermal involvement by monotonous, intermediate- to large-sized diffuse sheets of centroblasts and immunoblasts, large neoplastic cells with round oval nuclei, vesicular chromatin and prominent nucleoli, tumor necrosis may be observed, high mitotic activity with Ki-67 proliferation index exceeding 70% in most cases [9].

In a study documenting the clinical characteristics of 40 patients with PDCLBCL-LT (15 men, 25 women, mean age of 79 years, range: 46-98 years); in 32 cases, the lesions were located on the legs, two on the back, one on the head and neck, three on the trunk, and two in multiple sites. Twenty patients presented with a single lesion, and the other 20 presented with multiple lesions. During their five-month follow-up, they showed a five-year survival rate of 61.7% and a 10-year survival rate of 0%, indicating a poor prognosis, with a median survival of 12.5 months [10]. The clinical progression of a cutaneous B-cell lymphoma differs significantly from that of a cutaneous T-cell lymphoma. However, the atypical clinical evolution observed in this case - occurring in a young adult male and initially confirmed histopathologically under the suspicion of a T-cell lymphoma, but eventually developing specific clinical and immunohistochemical features consistent with a B-cell cutaneous lymphoma - highlights the potential for misclassification and the need for a thorough diagnostic process. This raises the question of a possible initial CD20 negativity. This is consistent with reports in the international literature, which have documented CD20-negative B-cell cutaneous lymphomas in approximately 1-2%, or even up to 3% of cases, with a noted association with chemoresistance and poor disease prognosis [5-7]. As we observed, indicators of poor prognosis translated into an ineffective treatment response.

For aggressive forms of DLBCL-LT, systemic therapies are recommended due to the poor prognosis and high relapse rates associated with this subtype. Based on recent cohort data, CHOP and R-CHOP (CHOP combined with rituximab) are considered the mainstay treatments, offering the longest time to subsequent treatment (TTNT) among systemic options. Rituximab monotherapy may also be considered in select cases [11]. However, despite adequate treatment in our patient, including autologous stem cell transplantation, there was no complete response. This could be related to the atypical presentation of the case and the fact that, as reported by other authors, the absence of CD20 at the beginning is associated with a poor prognosis.

## Conclusions

PCDLBCL-LT requires a multidisciplinary approach and individualized therapies to reduce relapse rates. Although immunohistochemistry remains key for accurate diagnosis, there are reported cases in which CD30 expression is initially absent. In this case, the evolution from a CD20-negative, CD30-negative profile during the initial diagnosis of mycosis fungoides to subsequent biopsies with a clear immunohistochemical profile (CD20+, MUM1+, and BCL2+) and nodular clinical presentation supports a final diagnosis of PCDLBCL-LT. This raises the possibility of either coexistence of both entities - mycosis fungoides and PCDLBCL-LT - or an early CD20-negative expression pattern, aligning with rare, atypical presentations in young male patients with generalized lesions outside the lower extremities and rapidly progressive disease.
